# Multicystic encephalopathy: an ultimate manifestation of ischemic-hypoxic injury

**DOI:** 10.4322/acr.2024.517

**Published:** 2024-09-27

**Authors:** Vielka Fernandez Aragones, Amilcar Castellano-Sanchez, Gabriel Chamyan, Darline Santana-Acosta

**Affiliations:** 1 Mount Sinai Medical Center, Arkadi M. Rywlin Department of Pathology and Laboratory Medicine, Miami Beach, FL, USA; 2 Florida International University, Herbert Wertheim College of Medicine, Department of Pathology, Miami, FL, USA; 3 Nicklaus Children's Hospital, Department of Pathology, Miami, FL, USA; 4 Nicklaus Children's Hospital, Pediatric Cardiac Critical Care, Miami FL, USA

**Keywords:** Autopsy, Brain, Hypoxia, Infant, Newborn

## Abstract

Multicystic encephalopathy is a rare neurological finding characterized by the appearance of multiple cystic or cavitary lesions as the result of repetitive episodes of hypoxic-ischemic injury in neonates and infants. We present a rare case of multicystic encephalopathy in a 3-month-old male, born at 34 weeks with Tetralogy of Fallot and multiple comorbidities. Gross examination of the brain during the autopsy revealed multiple irregular cystic lesions and distortion of the brain parenchyma. This case report highlights the uniqueness of multicystic encephalopathy and offers an extensive review of the existing literature, including etiology, clinical presentation, and histopathologic findings.

## INTRODUCTION

Multicystic encephalopathy was first recognized as a gross finding in 1954 by Wolf and Cowen^1^ while reviewing a series of deaths in early infants associated with cerebral atrophy. This entity is characterized by multiple areas of necrosis resulting in cystic cavities or spaces of varying sizes, located generally in the cerebral matter. Communication of these cavities with the ventricular system is not present.^2^ These cavities are usually the byproduct of recurrent prenatal or perinatal hypoxic episodes.^3^ Other causes have been described, such as encephalitis by cytomegalovirus and herpes virus,^4,5^ lactic acidosis due to congenital metabolic disorders,^6^ and abusive head trauma.^7^

We present the case of a 3-month-old male born at 34 weeks with multiple comorbidities, including Tetralogy of Fallot, tracheoesophageal fistula, and duodenal atresia. These conditions led to a prolonged hospital course, predisposing the patient to ischemic-hypoxic injury, among other possible hospital-associated complications. This case report highlights the rarity of multicystic encephalopathy and offers a comprehensive review of the existing literature on this neurological entity. This case report was conducted by CARE guidelines for case reports.

## CASE REPORT

A 34-week-old male patient was born to a 26-year-old multigravida woman. The mother was admitted for a cesarean section due to a twin pregnancy. The mother had adequate prenatal care and no history of teratogenic exposures to alcohol, tobacco, or prescription drugs. Prenatal screening for syphilis, hepatitis, rubella, HIV, and herpes were negative. The twin corresponding to this case report is a male infant with a birth weight of 1,900 g (18.1 percentile). Apgar scores were 6 and 8 at 1 and 5 minutes, respectively, and amniotic fluid was clear.

The infant was prenatally diagnosed with Tetralogy of Fallot and immediately presented with increased work of breathing and respiratory failure after birth, requiring intubation and one dose of surfactant. A double bubble sign was present on ultrasound, suggesting duodenal atresia. An orogastric tube could not be passed through the stomach, and an X-ray confirmed a blind upper esophageal pouch. These findings confirmed a tracheoesophageal fistula diagnosis, and the infant underwent surgical repair two days after birth.

Subsequently, his hospital course was complicated by esophageal pouch perforation and necrotizing enterocolitis, as well as left pulmonary artery coarctation requiring stent angioplasty. Seventy-four days after birth, the patient went into cardiopulmonary arrest, requiring more than 60 minutes of cardiopulmonary resuscitation (CPR). The infant was placed on extracorporeal membrane oxygenation (ECMO), and a video electroencephalograph (EEG) showed intermittent epileptic spikes. Neurologic examination at that time revealed bilateral asymmetric, non-reactive pupils. A brain computerized tomography (CT) performed seventy-nine days after birth showed findings concerning for diffuse cerebral hypoxic-ischemic injury. Recurrent pneumothoraces with multiple chest tube placements further complicated his hospital course. ECMO was discontinued at this time. 

Eighty days after birth, the patient developed pneumoperitoneum, which required emergent exploratory laparotomy and extensive lysis of adhesions along with small bowel resection and drainage of a peritoneal abscess. Antibiotic therapy at that time included Ampicillin, Meropenem, and Micafungin. Subsequently, an enterostomy was performed. Throughout his clinical course, the patient continued to require vasoactive medications and remained intubated on mechanical ventilation.

Throughout this prolonged admission, persistent hyperbilirubinemia was present, with a total bilirubin of 23.6 mg/dL (reference values: <2.0 mg/dL). He also developed elevation of transaminases with Alanine aminotransferase (ALT) and Aspartate aminotransferase (AST) values of 182 IU/L and 340 IU/L, respectively (reference values ALT: 12-45 IU/L, AST 9-80 IU/L). Infection with cytomegalovirus (CMV) was treated one hundred and five days after birth. Due to these laboratory findings and an inability to feed the patient enterally, a decision was made to re-anastomose his bowel. Subsequently, bleeding from the ostomy site prompted surgical removal of it along with bowel re-anastomosis that brought the patient into critical conditions following this procedure. Shortly after that, the patient developed hemorrhagic shock due to suspected coagulopathy from liver dysfunction and severe abdominal distention with multiorgan compromise. He went into cardiac arrest and despite resuscitative efforts, a return to spontaneous circulation was not achieved. The patient expired three months and twenty days after birth.

## AUTOPSY FINDINGS

On external examination, the patient’s body was well developed and well-nourished, appearing to be the stated age of three months, weighing 5,090 g, and measuring 54 cm from crown to heel, 38 cm from crown to rump, and 16 cm from rump to heel. An endotracheal tube was in place, and IV lines were present on both upper extremities. The skin was diffusely jaundiced. Mild edema was diffusely present on both the trunk and the extremities. A chest tube was present on the left side of the thorax, along with a well-healed scar on the right thorax, consisted with prior chest tube placement. Routine anthropometric measurements were within the normal reference range for age.^8^ The pupils measured approximately 0.6 cm on each side, and the sclerae were mildly icteric.

**Figure 1:**
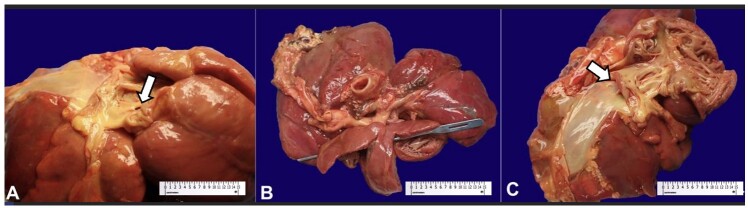
Gross images of the heart. **A –** Defect in the foramen ovale; **B –** Ventricular septal defect; **C –** Gross images of the heart showing overriding aorta.

Examination of the cardiovascular system revealed a heart that weighed 27 g (reference value: 23 g^8^) with a 0.1 cm defect located in the foramen ovale. A ventricular septal defect (VSD) was present. Hypertrophy of the right ventricle, along with stenosis of the pulmonary valve and right ventricle outflow tract were present, and the aortic valve was overriding (Figure 1). Hypoplasia of the left atrium was noted. Microscopic examination revealed foci of fibrosis and wavy fibers scattered through the myocardium.

**Figure 2:**
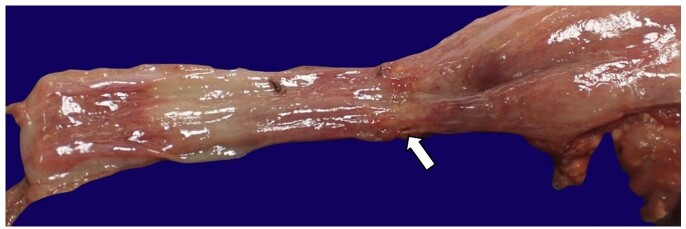
Gross image of the esophagus showing mild dilation proximal to repair of tracheoesophageal fistula.

The lungs were lobated and congested, with the right lung weighing 41 g and the left lung weighing 50 g (reference value: right, 35 g and left, 30 g)^8^ with diffuse intra-alveolar hemorrhage and consolidation with focal abscess formation. Adhesions to the right and left chest wall and diaphragm were present, and evidence of surgical repair was noted on the distal portion on the left side of the trachea (Figure 2).

The esophagus was patent, with gross and microscopic evidence of surgical repair and mild dilation proximal to the repair. The peritoneum was dry, and examination of the abdominal cavity revealed diffuse loop-to-loop and peritoneal adhesions with hematoma and multiple anastomosis sites in both small and large intestines. Capsular adhesions were present in the liver, which weighed 22.1 g (reference value: 14 g)^8^ as well as dark green discoloration. Severe cholestasis, dilated sinusoids, and atrophy of hepatocytes were microscopically identified. The kidneys showed medullary congestion with no significant microscopic findings. The spleen was congested, weighing 21.5 g (reference value: 14 g),^8^ and showed capsular adhesions.

**Figure 3:**
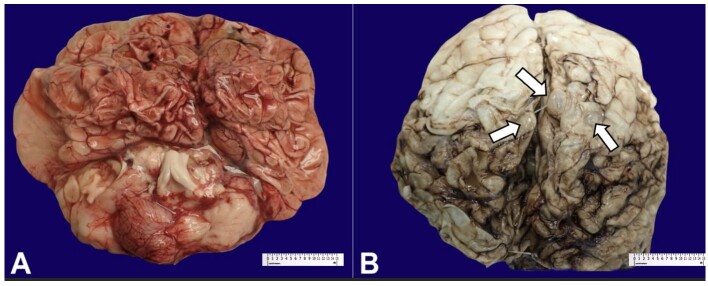
**A –** Gross image of fresh brain showing atrophic gyri and architectural disarray; **B –** Gross images of fixed brain showing flattened cerebrum with thin and congested leptomeninges along with small vesicles (white arrows).

The patient’s brain weight in the fresh state was 217 g (expected brain weight according to chronological age: 516 g)^8^. The brain showed collapsed architecture with atrophic gyri. The midbrain was not identified during the gross examination (Figure 3). Examination during the fixed state showed a flattened cerebrum and thin and congested leptomeninges (Figure 3). The two anterior thirds of the hemispheres were pale and flattened with small vesicles. The latter one-third of both cerebral hemispheres showed multiple irregular, soft areas. No hemorrhages, masses, or other discrete lesions were present apart from those already described. The corpus callosum was unremarkable, and no adhesions were identified between the hemispheres. Maximum thickness of the hemispheres ranged from 1 to 1.1 cm.

**Figure 4:**
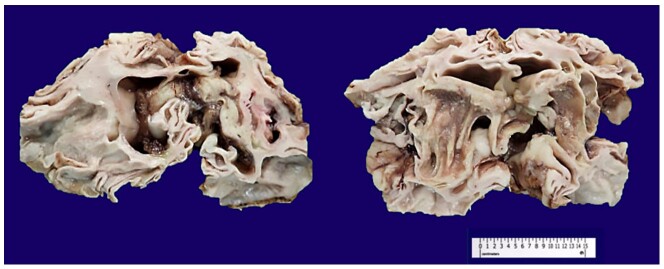
Coronal sections of the brain revealed the presence of multiple cavitary spaces, with almost complete replacement of gray and white matter.

Examination of the cerebrum’s undersurface showed the presence of optic chiasm and the posterior portion of the second cranial nerve. Additional cranial nerves or vessels were not identified due to diffuse fragmentation. Coronal sections of the brain revealed the presence of multiple cavitary spaces, with almost complete replacement of gray and white matter (Figure 4).

These lesions extended bilaterally from the frontal to the occipital lobes, with rare portions of preserved white matter in between. No hemorrhages were observed within these spaces. Bilateral cavitation of the basal ganglia was noted.

The ventricular system was only noted on the frontal horns of the lateral ventricle without any hemorrhages, collections, or masses. Midline distortion was noted due to the cavitary spaces. Sectioning through the brainstem and cerebellum revealed asymmetry of the cerebellar hemispheres, with the left hemisphere almost doubling the size of the right one. The dentate nucleus was not readily identified upon gross examination. Brown linear opacifications were noted in the medulla oblongata, extending down to the superior segment of the cervical spinal cord.

**Figure 5:**
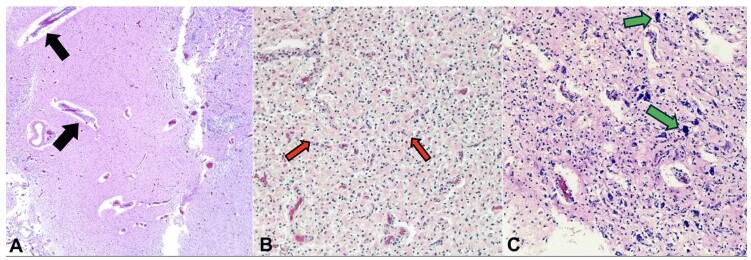
Photomicrographs of the brain showing. **A –** angiogenesis (black arrow); **B –** reactive astrocytes (red arrow); **C –** microglia and calcifications (green arrow), all evidence of infarction in various stages of evolution (A, B and C **–** H&E, 2.5x, 10x).

**Figure 6:**
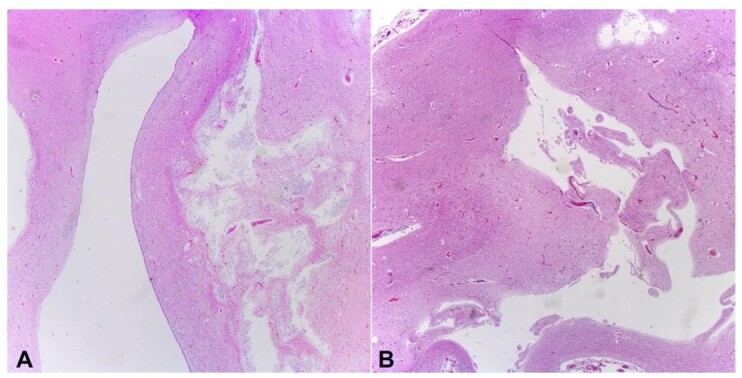
Photomicrographs of the brain. **A** and **B** – show the presence of multiple cavitary spaces in the brain parenchyma, with almost complete replacement of gray and white matter (A **–** H&E, 2x; B **–** H&E, 5x).

Histopathologic examination of the brain showed various stages in the evolution of cerebral infarction with areas of neutrophilic infiltrates along with diffuse vacuolation and microhemorrhages. Replacement of the cerebral parenchyma by macrophages, proliferating blood vessels, and reactive astrocytosis (Figure 5) were noted within the already described cavitary spaces (Figure 6). These massive changes were irreversible. Fragments of the choroid plexus, portions of ependymal lining, and preserved areas of white matter were identified.

Mild neuronal loss was noted in the dentate nucleus, as well as the presence of an external granular layer in the cerebellar foliae, as expected for the gestational age of the patient. Multifocal Purkinje cell loss and vascular congestion were significant findings. Sections of the brainstem also showed areas of infarction at multiple levels and at various stages of evolution.

## DISCUSSION

Multicystic encephalopathy (MCE) is characterized by the presence of cystic or cavitary degenerative lesions, generally located in the cerebral gray matter and the basal ganglia. In rare cases, these lesions may involve the white matter and the brain stem.^9^ Different terms have been used to describe this entity like multilocular encephalomyelomalacia, pseudoporencephaly, polyporencephaly, and encephalodystrophia.^2^ These lesions usually result from recurrent ischemic hypoxic injury in the third trimester of pregnancy or in the perinatal period. The changes associated with MCE are irreversible, and the most commonly accepted hypothesis is that they occur in the context of multiple episodes of hypoxia and ischemia alternating with episodes of reperfusion and re-oxygenation.^9^ If the insults occur before 24 weeks of gestation, cytoarchitectural and gyral disarray develops instead of MCE because the cortical plate neurons are in the last migration phase.^10^ Our patient had a hospital course complicated by multiple comorbidities, compromising oxygen supply to the central nervous system while undergoing extensive surgical procedures, which also increased his susceptibility to ischemic episodes alternating with episodes of reperfusion.

The cavitary lesions are formed due to liquefactive necrosis of the injured areas. This can occur in the setting of decreased oxygen uptake, like in carbon monoxide poisoning or asphyxia, or in cases of disruption of cerebral blood flow secondary to hypovolemic shock.^11^ Abusive head trauma has also been described as a precipitant factor for multicystic encephalopathy due to the hypoxia caused by compression of the vascular system. ^12^ The location of the lesions generally does not follow any specific vascular territory. In our case, the MCE histopathologic findings were observed in extensive areas of the brain parenchyma without a definitive pattern, extending through the medulla oblongata.

MCE is a rare occurrence in most hypoxic-ischemic insults in infants, which could indicate that there are unknown factors that could lead to the development of these lesions. Altered blood-brain barrier permeability caused by hypoxia leads to edema, venous congestion, and acidosis in the surrounding tissue. This cascade of events finally leads to the breakdown of myelin and the disruption and damage of white matter.^13,14^ One reported inciting factor is viral encephalitis, mostly due to cytomegalovirus or herpesvirus. This results in cytotoxic damage and subsequent inflammation in the affected areas.^15,16^ In our case, infection with cytomegalovirus could have been the tipping point in a long series of ischemic insults to the brain parenchyma, leading to irreversible damage. Other infectious entities associated with MCE include congenital toxoplasmosis, congenital syphilis, *E. coli*, Western equine encephalitis virus, and *H. influenzae*.^17-19^ Maternal exposure to carbon monoxide or butane as well as trauma, anemia, and preeclampsia have also been linked to MCE.^20^

Some inborn errors of metabolism have been described in association with MCE, like pyruvate dehydrogenase deficiency.^21^ Another associated condition is homozygous methylenetetrahydrofolate reductase mutation, which causes the accumulation of homocysteine, an amino acid with a neurotoxic sulfur group, leading to neuronal loss.^6^ Screening for these conditions in suspected cases is essential for diagnosis, and prompt treatment can improve neurological symptoms. Another precipitant factor for the development of MCE is abusive head trauma. Victims of this type of injury can quickly present with symptoms concerning cerebral edema, subdural hematoma, and subsequent increased intracranial pressure.^22^ Hypoperfusion of the brain secondary to shock or injury of the neck vasculature can also occur. A detailed physical examination and history, fundoscopic examination for retinal hemorrhages, and skeletal survey can help unveil abuse as the cause of MCE.^23^

Imaging techniques like cranial ultrasound play an essential role in identifying the cystic lesions. They should be performed in the diagnostic workup of infants with a history of intracranial infection, intracerebral hemorrhage, or asphyxia.^24^ This technique has the advantage of being low-cost, non-invasive, and does not expose the patient to unnecessary radiation. Serial cranial ultrasound has proven to be accurate in the measurement of cystic lesions and accessing the need for therapeutic management like shunts.^24^

The appearance of MCE is usually the last event in a cascade of changes caused by hypoxic-ischemic injury, resulting in irreversible neurological damage. Patients usually develop spastic quadriparesis, dementia, and ultimately, death.^9^

## CONCLUSIONS

MCE is a rare autopsy finding associated with prolonged, repeated episodes of hypoxic-ischemic injury in the perinatal and postnatal period, usually affecting the cerebral gray matter, the underlying white matter to some extent, and the basal ganglia. Due to the destruction of nervous tissue via necrosis, these findings are irreversible and lead to severe neurological signs and symptoms such as seizures, spastic quadriparesis or quadriplegia. The condition eventually results in death in most cases. Serial cranial ultrasound and computerized tomography can help guide the diagnosis in suspected cases. In our case, there were findings of intermittent epileptic spikes on EEG because of diffuse ischemic hypoxic injury, which imaging helped identify. This led to irreversible brain parenchyma damage, most likely resulting in severe neurological had the patient survived. Several risk factors have been associated with this condition, like viral encephalitis, abusive head trauma, and severe inborn errors of metabolism. Prognosis is poor once the lesion is identified since MCE is the result of devastating episodes of hypoxia and ischemia alternating with episodes of reperfusion and re-oxygenation, leading to irreversible neuronal loss and architectural disarray.
